# Health literacy interventions in adult speech and language therapy: A scoping review

**DOI:** 10.1111/hex.13878

**Published:** 2023-09-25

**Authors:** Verna B. McKenna, Órla Gilheaney

**Affiliations:** ^1^ Discipline of Health Promotion, School of Health Sciences University of Galway Galway Ireland; ^2^ School of Linguistic, Speech & Communication Sciences Trinity College Dublin Dublin Ireland

**Keywords:** health communication, health information, health literacy, patient information, scoping review, speech and language therapy

## Abstract

**Background:**

Reduced health literacy can negatively impact care seeking, satisfaction with care and overall health outcomes. These issues are particularly common among people living with communication difficulties who are seeking care from speech and language therapists (SLTs). As such, the SLT must be aware of and sensitive to health literacy needs within their clinical practice, proactively adapting materials and resources to the health literacy needs of their patients. Despite this required core competency, little is known about the health literacy interventions used by SLTs when working with adult patients, and as such, there is limited and unclear guidance for the practicing clinician, leading to potentially suboptimal care delivery.

**Objectives:**

To explore the characteristics of health literacy interventions discussed in the literature for use by SLTs with adult patients.

**Search Strategy:**

PubMed, CINAHL, Web of Science and The Cochrane Database of Systematic Reviews were searched. Conference proceedings of the annual scientific meetings of the European Society for Swallowing Disorders and the Dysphagia Research Society were also searched. Grey literature was searched via the Open Grey database and, hand‐searches of reference lists from included studies were conducted by both authors.

**Inclusion Criteria:**

Published and unpublished research investigating health literacy interventions provided by qualified SLTs providing care to adult patients in any setting for any speech and language related concerns. No language, geographic, study design or publication date limitations applied. Eligible participants in these studies were classified as: (1) patients and (2) professionals.

**Data Extraction and Synthesis:**

Data were charted in accordance with guidelines from the Joanna Briggs Institute and the PRISMA ScR (Preferred Reporting Items for Systematic Reviews and Meta‐Analyses extension for Scoping Reviews) independently by both authors.

**Main Results:**

A total of 1112 potentially eligible studies were identified in the initial search, with 15 studies ultimately included in the synthesis. Study design and quality varied significantly. Most explored basic functional health literacy or narratively described core components of health literacy, which an SLT should understand, without employing an investigative research design.

**Discussion:**

Limited research has been conducted on the use of health literacy interventions within adult speech and language therapy practices. This finding is significant as SLTs regularly work with people living with communication problems, and therefore, addressing health literacy should be a core tenet of service delivery.

**Conclusion:**

There is a need for valid, reliable and rigorous investigations of health literacy interventions within speech and language therapy to ultimately improve future patient access to and benefit from the care provided.

**Patient or Public Contribution:**

Patient public involvement in review studies is an emerging area. Due to resource issues, it was not possible to include this element in this study.

## 1 INTRODUCTION

Many people experience difficulties accessing and using health information and services, which can negatively impact their health outcomes. Health literacy is a relational concept defined as the personal knowledge and competencies that enable people to access, understand, appraise and use information and services in ways that promote and maintain good health and well‐being for themselves and those around them.^1^ These are mediated by organizational structures and the availability of resources. Thus, health care providers and their associated organizations are a key source of such mediation. Health literacy is a complex concept, comprising a set of personal competencies, but dependent on the characteristics of the health care systems and services that can facilitate or hinder the usage of health information for health‐related decisions.^2^

The link between health literacy and health outcomes is well established and includes greater mortality and poorer overall health status, increased hospitalizations and emergency care use when health literacy levels are low.^3^, ^4^, ^5^, ^6^, ^7^, ^8^, ^9^ This has been especially underlined in conditions, such as asthma, diabetes and obesity.^3^, ^5^, ^6^ Health literacy is also found to be a stronger predictor of outcomes than race/ethnicity, income and education.^3^

The three‐level model of health literacy, comprising functional, interactive and critical levels of health literacy, provides a useful framework for categorizing the focus of various health literacy interventions.^10^ Functional health literacy is based on the communication of factual information and how to use the health care system. Interactive health literacy focuses on the development of personal skills in a supportive environment so that individuals are better able to act independently in regard to their health care. Finally, critical health literacy reflects more advanced cognitive skills, which, together with social skills, can be applied to critically analyse information, and to use this information to exert greater control over life events and situations.^10^ It stands to reason that people who experience communication disorders potentially face even greater challenges. Individuals with speech, language and/or hearing disorders are vulnerable to low health literacy as they are likely to encounter challenges interpreting and applying health information.^11^, ^12^ This population also reports that difficulties in communicating with medical professionals often result in stressful and difficult experiences.^13^ Despite this potential for misunderstandings and therefore poorer patient outcomes, there is a dearth of research literature on the health literacy needs and supportive measures for people with communication disorders who attend speech and language therapy. Disorders such as aphasia, by their very nature, make communication, for example, between patient and provider very difficult, which, in turn, can negatively impact the ability of the patient to access and understand important health information and to take part in health care‐related decision‐making.

In light of this increased risk for lower levels of health literacy and therefore higher levels of miscommunications between clinicians and patients, this scoping review was framed by the following research question (RQ): What health literacy interventions have been discussed in the literature for qualified speech and language therapists (SLTs) who are providing care to adult patients aged 18 years and above in any setting for any speech and language related concerns? This scoping review aimed to explore the characteristics of health literacy interventions that have been discussed in the literature for use by SLTs with adult patients.

## 2 METHODOLOGY

### 2.1 Overview of study design

The fundamentals of the methodology were based on Arksey and O'Malley's five‐step framework, which included (1) defining the RQ, (2) identifying relevant studies, (3) study selection, (4) data charting and (5) collating, summarizing and reporting the findings.^14^ The study was also informed by the Preferred Reporting Items for Systematic Reviews and Meta‐Analyses extension for Scoping Reviews (PRISMA‐ScR) minimum set of guidelines as outlined by Tricco et al.^15^

### 2.2 Materials and methods

#### 2.2.1 Eligibility criteria

The Population, Concept and Context (PCC) framework was used to develop the RQ, guide the search strategy and identify specific study characteristics eligible for inclusion in the scoping review. This framework, applied to the current scoping review, is set out in Table 1 below.^16^

**Table 1:** Population, concept and context of review.

	Patients	Professionals
Population	Adult patients aged 18 years and above	SLTs who are qualified to practice and who are providing care to adult patients aged 18 years and above in any setting for any speech and language related concerns
Concept	Health literacy interventions Patient communication Patient information	Health literacy interventions Patient communication Patient information
Context	Adult patients who are attending SLTs in any setting for any speech and language related concerns	SLTs who are providing care to adult patients in any setting for any speech and language related concerns

Abbreviation: SLTs, speech and language therapists.

Eligibility criteria for studies were as follows: published and unpublished research investigating health literacy interventions provided by qualified SLTs who are providing care to adult patients (aged 18 years and above) in any setting for any speech and languag related concerns. Articles that did not explicitly refer to health literacy were discussed by both authors in the context of the study objectives. For example, if the objectives of an article focused on improving the readability of material to increase patient understanding then these were deemed suitable (related to access and understanding). One of the authors has extensive expertise in health literacy research, which helped in the decision‐making. No language, geographic, study design or publication date limitations were applied. Eligible participants in these studies were classified as: (1) patients: adult patients aged 18 years and above, who are attending speech and language therapy in any setting for any speech and language related concerns; and (2) professionals: SLTs who are qualified to practice and who are providing care to adult patients aged 18 years and above in any setting for any speech and language related concerns. This classification was used to assist in the identification of interventions across various settings as well as those that focused on SLT professionals only (raising awareness of health literacy) and those that directly targeted meeting the health literacy needs of patients.

#### 2.2.2 Information sources, search and selection of sources of evidence

A search strategy that incorporated filters and MeSH, and key‐text terms was designed by both authors (see Supporting Information for search string). Databases searched by one author (O. G.) from inception to October 2021 were: PubMed, CINAHL, Web of Science and The Cochrane Database of Systematic Reviews. No limits regarding publication date, language or country of origin were applied to the search. Conference proceedings of the annual scientific meetings of the European Society for Swallowing Disorders (published in *Dysphagia*) (2011–2021) and the Dysphagia Research Society (published in *Dysphagia*) (1992–2021) were also searched. Grey literature was searched via the Open Grey database. Finally, hand‐searches of reference lists from included studies were conducted by both authors. Author contact was conducted, via email. in the case of lack of access to articles or missing data, as required.

Potentially eligible studies were exported into Covidence,^17^ with subsequent duplicate deletion by the primary author. Both authors independently screened the titles and abstracts of 50% of potentially eligible studies and irrelevant articles were removed. Any articles where a clear exclusion/inclusion decision could not be easily made were retained in a ‘consensus needed’ file. Both authors then discussed these in the context of the a priori eligibility criteria. This allowed consensus to be reached on articles to include/exclude.

#### 2.2.3 Review process and data charting

Following this initial screening of titles and abstracts, both authors independently reviewed all of the full‐text articles. After reviewing, both reviewers discussed the decisions made and verified the screening accuracy. The data charting form was developed in accordance with guidelines from the Joanna Briggs Institute^16^ and the PRISMA ScR,^15^ with both authors independently charting data from each included study, and subsequently cross‐checking and reconciling on ‘maybes’ until 100% agreement was reached. Data were charted from included studies across a range of parameters (Table 2).

**Table 2:** Data items and specifics.

Data item	Data specifics
General study details	Title
	Lead author contact details
	Country in which the study conducted
	Aim of study
	Study funding sources
	Possible conflicts of interest for study authors
Methodology	Study design
	Start date/end date
	Population description
	Inclusion and exclusion criteria
	Method of recruitment of participants
	Total number of participants
	Health literacy levels
	Data items
	Data collection methods
	Data analysis,
Results of Study	Quantitive data
	Qualitative data
	Synthesis of data
Conclusion	Conclusion of study

Microsoft Excel was used to extract the data from each of the 15 articles with data charted and sorted according to key issues and themes.^14^ Data were charted using MS Excel to elicit relevant data relating back to the RQ and aims and objectives of this scoping review. Table 2 sets out the headings initially used to organize and chart data.

Nutbeam's framework, delineating the three levels of health literacy: functional, interactive and critical, was used to categorize the health literacy interventions identified in the final 15 articles.^10^ This was undertaken by the first author (VBMcK) who has expertise in health literacy and subsequently discussed with the second author.

#### 2.2.4 Critical appraisal of individual sources of evidence

Although scoping reviews do not typically include quality or appraisal elements,^16^ the authors drew on the PRISMA‐ScR guidelines to guide the critical appraisal of this study.^18^ This was deemed an important element in the context of health literacy research where there can be variance in the types of intervention used. Furthermore, omission of this step may often be considered a limitation in scoping reviews due to challenges posed in the context of making recommendations for future practice and/or research, as well as in the detection of gaps in knowledge.^19^ Therefore, critical appraisal of included studies was conducted to explore the relative strengths and weaknesses of available studies on this topic, while also contributing to recommendations for advancing future research. Due to the heterogeneity of designs within included studies, a range of critical appraisal tools were used to ensure that appropriate reference points were assessed (e.g., cross‐sectional, quasiexperimental literature reviews, opinion pieces, qualitative and case study designs).^20^, ^21^, ^22^, ^23^, ^24^, ^25^ No studies were excluded based on the outcome of the critical appraisal process.

#### 2.2.5 Narrative synthesis of results

Charted data was synthesized based on the guidelines of Arksey and O'Malley, using narrative methods and descriptive statistics via Microsoft Word and Excel.^14^ The collation of findings began with a descriptive summary of each of the included articles, allowing for identifying any themes that were not included in the original categories outlined for charting (Table 2). The narrative synthesis, described by Popay et al. as ‘using words and text to summarize and explain the findings of the synthesis’ (p. 26), aimed to contextualize the findings of the included literature relevant to the RQ.^26^ The findings are discussed under headings related to the study design and level of health literacy intervention (according to the Nutbeam framework outlined earlier). The methods used in the health literacy interventions were further delineated to include readability analysis and/or use of quality assessment tools relevant to both written and internet‐based materials.

## 3 RESULTS

### 3.1 Selection of sources of evidence

**Figure 1:**
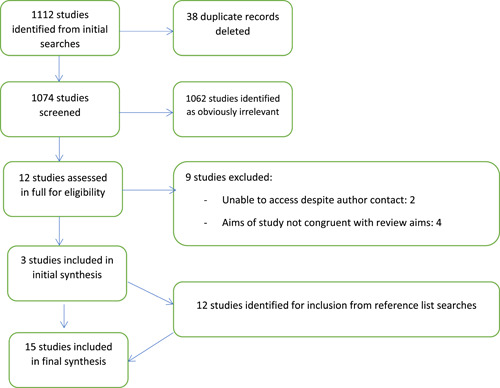
Preferred Reporting Items for Systematic Reviews and Meta‐Analyses flow diagram: A summary of the literature search and review process.

In total, 1112 records were identified in the initial search (see Figure 1) and exported first to Zotero,^27^ then Covidence.^17^ Covidence was used to automatically delete 38 duplicate records. Both authors then independently screened the titles and abstracts of 50% of the remaining 1074 studies each, with 1062 obviously irrelevant studies excluded at this stage. Full‐text review of 12 studies was independently conducted by both authors, with nine excluded due to various reasons such as nonalignment with the objectives of the scoping review. As such, three studies were initially included in the synthesis. Subsequent to reference list searches, a further 12 studies were deemed eligible, resulting in 15 studies being included in the scoping review synthesis.

### 3.2 Characteristics of sources of evidence

Table 3 and Supporting Information: Material A summarize the characteristics of the 15 studies included in the final analysis.

**Table 3:** Study characteristics.

References	Aims	Study design	Details of health literacy intervention
[28]	To examine the readability of audiology‐ and speech‐language pathology‐related consumer materials on the website of the ASHA. To report their findings relative to the reading grade‐level guidelines recommended by health literacy	Cross‐sectional study	The study addressed functional health literacy as it focused on the readability of audiology and speech and language related consumer materials. The readability findings were reported relative to the reading grade‐level guidelines recommended by health literacy experts
			*Readability tests/formulae used*: 1. FRE^29^ 2. F–K^30^ 3. FORECAST^31^
[32]	To assess the quality and readability of English language Internet information available for aphasia treatment	Cross‐sectional study	The study addressed functional health literacy by focusing on an assessment of the quality and readability of English‐language internet information available for aphasia treatment
			*Readability tests/formulae*: 1. FRE^29^ 2. Flesch–Kincaid grade level^30^ 3. SMOG^33^
			*Quality assessment tools* 1. HON certification^34^ 2. DISCERN^35^
[36]	To evaluate the readability and quality of English‐language Internet information related to vocal hygiene, vocal health and prevention of voice disorders	Cross‐sectional study	The study addressed functional health literacy through an evaluation of the readability and quality of English‐language Internet information related to vocal hygiene, vocal health and prevention of voice disorders
			*Readability tests/formulae* 1. FRE^29^ 2. Flesch–Kincaid grade level^30^ 3. SMOG^33^
			*Quality assessment tools* 1. HON certification^34^ 2. DISCERN^35^
[37]	To determine the appropriateness of current written educational materials for stroke survivors and their carers in terms of their readability, content and design	Qualitative study	This study addressed functional health literacy. It focused on readability/ability to understand levels of written stroke education material
			*Readability tests/formulae* 1. The REALM^38^ 2. RIX formula^39^ 3. Suitability Assessment of Materials^40^
[41]	To develop and validate an educational booklet to facilitate changes in dietary routines in patients with dysphagia	Cross‐sectional study	This study addresses the functional level of health literacy as it is focused on the development and validation of the informational booklet
[42]	Not explicitly stated	Opinion piece	The article discusses a number of different strategies to address written and verbal communication. These strategies address the functional level of health literacy. Interactive health literacy is also addressed through the lens of improving communication with health care professionals
[12]	Not explicitly stated	Opinion piece	The article discusses a number of different strategies to address health literacy in the context of communication disorders. Functional health literacy is addressed in the main body of the text and interactive health literacy is also addressed as a secondary point by discussing improving communication with health care professionals
[11]	To discuss various definitions and concepts related to health literacy To provide information on existing health literacy assessments To describe health literacy characteristics in populations identified as having low or inadequate health literacy To review language and communication studies related to health literacy. To propose theoretical frameworks for speech‐language and hearing professionals to become involved with health literacy research, assessments and interventions	Literature review	This was a discussion piece that addressed all areas of health literacy and proposed a conceptual approach to health literacy for SLTs. This piece discusses health literacy assessments including: the TOFLA,^43^ the S‐TOFLA^44^ and REALM^38^
[45]	To see whether following the guidelines of the *NHS Toolkit for Producing Patient Information* improved the readability of the leaflets in an SLT department	Quasiexperimental	This study addressed functional health literacy. It focused on revising current departmental leaflets using the NHS Toolkit for Producing Patient Information.^46^ It then tested the effect of these revisions on the readability scores of the text
			*Readability tests/formulae*: 1. FRE^29^ 2. Flesch–Kincaid grade level^30^ 3. NHS Toolkit for Producing Patient Information^46^
[47]	Not explicitly stated	Opinion piece	A number of different strategies are addressed in this piece, which focuses on raising SLTs awareness of both function and interactive health literacy. Regarding interactive health literacy, the article addresses communication between patient and provider with an emphasis on teach‐back, encouraging patients to ask questions and developing a culture that supports this. This article also provides information on steps SLTs can take to improve communication
[48]	To examine the readability of voice‐related PROMs with consideration to expert recommendations for optimal reading grade level	Literature review	The study addressed functional health literacy as it focused on investigating whether voice‐related PROMs developed and validated since 2011 met the recommendation by health literacy experts that such materials be written at a fifth‐to‐sixth‐grade reading level.
			*Readability tests/formulae*: 1. Coleman–Liau index^49^ 2. FORCAST^31^ 3. FOG^50^ 4. SMOG^33^ 5. FRE^29^
[51]	To trial technology/strategies that may maximize health literacy and client recall of clinical information To evaluate the outcomes of the technology/strategies employed from both the SLT's and the client/caregiver's perspective	Case study	Both functional and interactive health literacy were addressed. The study focused on combined strategies that can assist the client in managing their health condition and communicating effectively with the SLT
[52]	To examine the grade level and readability of commonly used English‐language voice‐related PROM questionnaires and to compare this data to existing information about average reading levels of English‐speaking adults living in the United States	Cross‐sectional study	Functional health literacy addressed. The study examined the readability of published questionnaire PROMs
			*Readability tests/formulae*: 1. FORCAST^31^ 2. FOG^50^ 3. FRE^29^
[53]	To examine the readability of several published patient PROM questionnaires for use with persons who stutter and to compare this data to existing information about average reading levels of English‐speaking adults living in the United States	Cross‐sectional study	Functional health literacy addressed. The study examined the readability of published questionnaire PROMs
			*Readability tests/formulae*: 1. FORCAST^31^ 2. FOG^50^ 3. FRE^29^
[54]	To examine the grade level and readability of published PROM questionnaires for persons with swallowing problems and to compare this data to existing information about average reading levels of English‐speaking adults living in the United States	Cross‐sectional study	Functional health literacy addressed. The study examined the readability of published questionnaire PROMs
			*Readability tests/formulae*: 1. FORCAST^31^ 2. FOG^50^ 3. FRE^29^

Abbreviations: ASHA, American‐Speech‐Language‐Hearing Association; F–K, Flesch–Kincaid; FOG, Fog index; FRE, Flesch Reading Ease; HON, Health On the Net; NHS, National Health Service; PROM, patient‐reported outcome measurement; REALM, Rapid Estimate of Adult Literacy in Medicine; SLT, speech and language therapist; SMOG, Simple Measure of Gobbledygook; S‐TOFLA, Short Test of Functional Health Literacy in Adults.

All studies were published in English, with 73.3% (*n* = 11) conducted in the United States (see Table 3). Most studies were cross‐sectional studies (46.66%; *n* = 7) or opinion pieces (20%; *n* = 3).

Participants, patient information leaflets and clinical assessment tools were studied in the articles included here. A small cohort (*n* = 23) of patients were included, in addition to 15 caregivers.^37^, ^51^ Due to the nature of the abstract‐only text, the authors here could not determine the exact numbers of patients/caregivers in Ferreira and Figueiredo‐Braga's^41^ mixed cohort (*n* = 6). Few SLTs were included (*n* = 8)^41^, ^51^ in conjunction with unspecified experts in clinical communication (*n* = 4).^41^ Patient‐directed information included 101 audiology‐related^28^ and 144 speech and language related leaflets or articles^28^, ^45^ in addition to 128 websites of varying origin/focus.^32^, ^36^ Patient‐reported outcome measurement tools were reviewed across four studies, with 34 assessments considered in total (Voice: *n* = 20; Stuttering: *n* = 10; Swallowing: *n* = 4).^48^, ^52^, ^53^, ^54^

Readability refers to the ease with which a person can read and understand written information.^55^ Various readability software tools can be used to analyse how closely scores correlate with reading comprehension scores.^56^

The majority of studies, in this review, employed readability analysis using a variety of assessment measures with subsequent statistical exploration (*n* = 9; 60%).^28^, ^32^, ^36^, ^37^, ^48^, ^52^, ^53^, ^54^ Narrative discussion was common in a quarter of all included studies (*n* = 4; 26.6%).^11^, ^12^, ^42^, ^47^

Quality assessment tools provide a systematic method for judging the quality of patient information materials. These have been developed to address limitations in the evaluation of content or effectiveness.^35^

### 3.3 Interventions

Nutbeam's framework on health literacy levels was applied to the final 15 studies.^10^ Findings indicated that most studies addressed health literacy at a functional level only (*n* = 10; 67%) (see Table 3).

Opinion piece and literature review articles also focused on functional health literacy in the main, with some emphasis on interactive health literacy^42^, ^47^ a literature review by Hester and Stevens‐Ratchford,^11^ was the only article to discuss all three levels of health literacy (functional, interactive and critical) and their relevance for speech and language therapy.

The majority of health literacy interventions focused on readability analysis of existing patient information materials. In the main, these related to printed materials (*n* = 8, 53%) with a smaller number examining web‐based materials (*n* = 3, 20%)^28^, ^32^, ^36^: Opinion piece and literature review articles provided a broader focus on health literacy by discussing its relevance for the SLT, the importance of raising awareness of health literacy for SLTs and strategies for SLTs to employ to improve their communication techniques.^11^, ^12^, ^42^, ^45^, ^47^ One of these also addressed the research agenda for SLTs in relation to health literacy by highlighting the need to investigate the impact of communication disorders on health literacy and examining the relationship between existing health literacy assessments and speech‐language and hearing.^12^

### 3.4 Use of readability tests and formulae

The eight studies that assessed readability employed readability analysis with some variation in the types of reading tests used across the studies. The most commonly used was the Flesch Reading Ease.^54^

### 3.5 Quality assessment tools

There was also some variation in the use of quality assessment tools within the eight studies included with different tools used in the studies, which included Health On the Net certification, DISCERN, NHS Toolkit for Producing Patient Information and the Suitability Assessment of Materials (see Table 3).^28^, ^32^, ^36^, ^37^, ^45^ One study did a comparison of materials before and after applying the readability analysis and making changes.^45^

### 3.6 Study conclusions

Conclusions were overall homogeneous in their statements. Several studies commented on the lack of information on health literacy within the field of speech and language therapy while flagging the need to strengthen the evidence base in this area.^11^, ^12^ With regard to publicly available consumer information on speech and langugae topics, the readability, relevance and accessibility of these resources was typically low, with authors hypothesizing that this information in its current form would be of limited value to those with low levels of health literacy. Therefore, misinterpretations and miscommunications could occur, with implications for patient and caregiver outcomes.^28^, ^32^, ^36^, ^37^, ^41^, ^47^ In reference to speech and language therapy assessments, authors typically concluded that patient‐reported outcomes focusing on voice, swallowing and stuttering do not fully comply with appropriate reading grade levels for patients with differing levels of health literacy.^48^, ^52^, ^53^, ^54^ A recurrent conclusion was the responsibility that clinicians have to ensure that the written information that they provide to patients or the assessments that they use with them are accessible and meaningful to patients and their caregivers^12^, ^28^, ^32^, ^37^, ^41^, ^45^, ^51^ (see Supporting Information: Materials B).

### 3.7 Critical appraisal of sources of evidence

As discussed, qualitative critical appraisal was conducted using a variety of study‐specific tools (see Supporting Information: Material C for full critical appraisal details). Study designs were highly heterogeneous, precluding meaningful comparison among included studies in most cases. With regard to the most common study design, cross‐sectional studies, the majority of these articles provided unclear information regarding the identification and management of potentially confounding factors, in addition to underlying difficulties in the appropriate and transparent description of study subjects.

## 4 DISCUSSION

This scoping review represents the first attempt at compiling and evaluating all available information regarding health literacy interventions used by SLTs working with adult patients. The findings of this study suggest that despite SLTs being specially trained to work with people experiencing communication difficulties and although they are often considered experts in facilitating such patients to interact as effectively as possible via any mode of communication, health literacy has been minimally considered within research in this field. SLTs should be considered a key profession for addressing professional health literacy in the context of communication with patients and users and the comprehensive dissemination of health‐related information to patients.^57^ In addition, when health literacy has been researched within the field of speech and language therapy, investigations have primarily focused on basic functional health literacy, often relying on narrative means of analysis, as opposed to more quantitative, structured or systematic methods. There was significant heterogeneity and variable quality of conduct within the 15 studies included in this scoping review, which greatly hindered the synthesis of findings. As such, limitations to validity and reliability are common, undermining the potential of research gathered here to meaningfully contribute to the professional evidence base and provide useful guidance for practicing clinicians.

The findings of this scoping review highlight an almost exclusive focus on functional health literacy, demonstrated through the application of readability formulae to written and/or web‐based patient information materials, as well as a paucity of attention to patient‐provider communication. While this ‘low‐hanging fruit’ approach is common across health services in general, it is crucial that providers recognize that health literacy extends beyond the ability to access websites, read pamphlets and follow healthy lifestyle advice.^1^ Instead, the focus should be on empowering patients to improve their ability to act independently and take responsibility for their own care needs. Empowering the person with a communication disorder to be able to participate independently in their communities should be the key focus of health literacy interventions in the field of speech and language therapy practice and research. It is also worth noting that the literature on self‐management of chronic illness and health literacy supports the importance of both the interactive and critical levels of HL as stronger predictors for successful self‐management than functional health literacy.^58^ It is not unreasonable to assume a similar outcome for the management of long‐term communication disorders.

It is also important to note that readability formulae cannot provide any information about comprehension beyond mathematical calculations of the elements that make up the sentences/phrases in the materials.^59^ Comprehension is also dependent on other factors including familiarity with the topic, self‐motivation, topic of interest, cultural competency, layout/design, appropriate use of language and tense of writing. As these elements were not typically measured by studies in this review, we cannot fully comment on the levels of understanding of the materials. Further research should include these elements as a guide for developing health literacy assessments and testing patient materials related to various communication disorders.^11^ Nonetheless, the findings are important as they demonstrate that readability levels fall short of recommended levels and so there is an increased risk that patients information needs 'are not been met. Another important point, noted in many studies, is that there is no ‘gold standard’ when it comes to readability assessments. Future research should include service users in the assessment of accessibility of patient materials as well as eliciting their recommendation for preferred mode of transmission.

Narrative literature review and opinion piece articles provided a broader discussion of health literacy that also emphasized the role of the SLT in supporting health literacy for their service users. These articles demonstrated that there is a burgeoning awareness of the relevance and importance of addressing health literacy in sppech and language practice, addressing the interactive level. However, this has not translated to practice and research. The lack of a universally accepted definition of health literacy is a likely contributor to studies that are not explicitly labelled ‘health literacy’ in the literature. Therefore, it is likely that there may be interventions underway that address broader issues of empowerment, supporting patients with communication disorders to thrive in the community. Although such work is aligned with critical health literacy these are not labelled as such in the literature. Therefore, locating these using a scoping review methodology is challenging. Increased emphasis on labelling health literacy to such studies can increase the visibility of positive outcomes associated with health literacy and provide important leverage for policy and funding opportunities to expand interventions that support and empower people with communication disorders to be able to participate fully and ‘live well’ in the health and community domains of their lives.^60^ Developing appropriate health literacy interventions for people with communication disorders is complex, addressing both the barriers posed by communication disorders as well as the many challenges already inherent in health communication.

### 4.1 Limitations

This study greatly adds to the professional knowledge base and acts as the first attempt to review all literature on health literacy interventions used by SLTs working with adult patients. However, as with all research, there are identified limitations. Despite rigorous attempts to design a sensitive search string, most articles that were ultimately included in the synthesis were identified via reference list searches of eligible articles. This was attributed to the significant diversity in the terminology used to describe health literacy interventions, which made it difficult for authors to identify articles in the initial systematic search. The unstandardized terminology used, coupled with the significant diversity within study design, conduct and publication method, complicates the gathering of all research on this topic and therefore the planning of clinical services which are informed by valid and reliable evidence. Therefore, future work focusing on the standardization of terminology, the development of rigorous means of implementation and evaluation of health literacy interventions in speech and language therapy, and the effective application of this knowledge to clinical practice is required. Another limitation is that this review did not include patients as partners in the review process.^61^ It is possible that including patient public involvement in developing the RQ and search strategy could have assisted us in capturing a broader range of studies.

Both authors did not independently review the entire set of titles and abstracts. Instead, each of the two authors reviewed 50% of each of these due to time constraints. However, all queries on title and abstract screening were addressed by both authors and a consensus was reached on inclusion and exclusion status.

## 5 CONCLUSIONS

This study demonstrated that there is limited research on the use of health literacy interventions within adult speech and language therapy practices. This finding was surprising, given that SLTs regularly work with people experiencing communication problems, and therefore, addressing health literacy is a core tenet of basic service delivery. In addition, this study found that where research in this area does exist, health literacy was often addressed in a superficial and unstandardized manner, focusing typically on narrative descriptions of functional health literacy, and therefore limiting the advancement of our evidence base in this area. Therefore, this scoping review has emphasized the need for valid, reliable and rigorous investigations of health literacy interventions within speech and langugae therapy to ultimately improve future patient access to and benefit from the care provided.

## AUTHOR CONTRIBUTIONS

Verna B. McKenna and Órla Gilheaney contributed equally to manuscript concept, production, and research conduct and analysis.

## CONFLICT OF INTEREST STATEMENT

The authors declare no conflict of interest.

## Supporting information

Supporting information.

Supporting information.

Supporting information.

Supporting information.

## Data Availability

Data sharing is not applicable to this article as no new data were created or analysed in this study.
