# Supplementary material for: Health literacy interventions in adult speech and language therapy: A scoping review
Source: Health Expect. 2023 Sep 25;27(1):e13878. doi: 10.1111/hex.13878 (PMC10726155; doi:10.1111/hex.13878)
Supplement: Supplementary file 1 — Supporting information. [file HEX-27-e13878-s001.docx]

(“Patient communication”[Title/Abstract] OR “Patient information”[Title/Abstract] OR “Health information”[Title/Abstract] OR “Consumer Health Information”[Mesh] OR “Information, Consumer Health”[Title/Abstract] OR “Health Information, Consumer”[Title/Abstract] OR “Health Information Exchange”[Mesh] OR “Exchange, Health Information”[Title/Abstract] OR “Exchanges, Health Information”[Title/Abstract] OR “Health Information Exchanges”[Title/Abstract] OR “Information Exchange, Health”[Title/Abstract] OR “Information Exchanges, Health”[Title/Abstract] OR “Medical Information Exchange”[Title/Abstract] OR “Exchange, Medical Information”[Title/Abstract] OR “Exchanges, Medical Information”[Title/Abstract] OR “Information Exchange, Medical”[Title/Abstract] OR “Information Exchanges, Medical”[Title/Abstract] OR “Medical Information Exchanges”[Title/Abstract] OR “Health Information Management”[Mesh] OR “Health Information Managements”[Title/Abstract] OR “Information Management, Health”[Title/Abstract] OR “Information Managements, Health”[Title/Abstract] OR “Management, Health Information”[Title/Abstract] OR “Managements, Health Information”[Title/Abstract] OR “Health literacy”[Mesh] OR “Literacy, Health”[Title/Abstract] OR “Literacy”[Mesh] OR Illiteracy[Title/Abstract] OR “Information literacy”[Mesh] OR “Information Literacies”[Title/Abstract] OR “Literacies, Information”[Title/Abstract] OR “Literacy, Information”[Title/Abstract] OR “Numeracy”[Title/Abstract] OR “Patient understanding”[Title/Abstract] OR “Patient appraisal”[Title/Abstract] OR “Health communication”[Mesh] OR “Communication, Health”[Title/Abstract] OR “Communications, Health”[Title/Abstract] OR “Health Communications”[Title/Abstract] OR “Medication adherence”[Mesh] OR “Adherence, Medication”[Title/Abstract] OR “Drug Adherence”[Title/Abstract] OR “Adherence, Drug”[Title/Abstract] OR “Medication Nonadherence”[Title/Abstract] OR “Nonadherence, Medication”[Title/Abstract] OR “Medication Noncompliance”[Title/Abstract] OR “Noncompliance, Medication”[Title/Abstract] OR “Medication Non-Adherence”[Title/Abstract] OR “Medication Non Adherence”[Title/Abstract] OR “Non-Adherence, Medication”[Title/Abstract] OR “Medication Persistence”[Title/Abstract] OR “Persistence, Medication”[Title/Abstract] OR “Medication Compliance”[Title/Abstract] OR “Compliance, Medication”[Title/Abstract] OR “Medication Non-Compliance”[Title/Abstract] OR “Medication Non Compliance”[Title/Abstract] OR “Non-Compliance, Medication”[Title/Abstract] OR “Drug Compliance”[Title/Abstract] OR “Compliance, Drug”[Title/Abstract] OR “Treatment Adherence and Compliance”[Mesh] OR “Therapeutic Adherence and Compliance”[Title/Abstract] OR “Treatment Adherence”[Title/Abstract] OR “Adherence, Treatment”[Title/Abstract] OR “Therapeutic Adherence”[Title/Abstract] OR “Adherence, Therapeutic”[Title/Abstract] OR “Treatment decision-making”[Title/Abstract] OR “Patient Participation”[Mesh] OR “Participation, Patient”[Title/Abstract] OR “Patient Involvement”[Title/Abstract] OR “Involvement, Patient”[Title/Abstract] OR “Patient Empowerment”[Title/Abstract] OR “Empowerment, Patient”[Title/Abstract] OR “Patient Participation Rates”[Title/Abstract] OR “Participation Rate, Patient”[Title/Abstract] OR “Participation Rates, Patient”[Title/Abstract] OR “Patient Participation Rate”[Title/Abstract] OR “Patient Activation”[Title/Abstract] OR “Activation, Patient”[Title/Abstract] OR “Patient Engagement”[Title/Abstract] OR “Engagement, Patient”[Title/Abstract] OR “rapid estimate of adult literacy”[Title/Abstract] OR REAL* OR “test of functional health literacy”[Title/Abstract] OR TOFL* OR “newest vital signs”[Title/Abstract] OR NVS* OR “Short assessment of health literacy”[Title/Abstract] OR SaHL*) AND (“Speech-Language Pathology”[Mesh] OR “Speech Language Pathology”[Title/Abstract] OR “Pathology, Speech-Language”[Title/Abstract] OR “Pathology, Speech Language”[Title/Abstract] OR “Language Pathology”[Title/Abstract] OR “Pathology, Language”[Title/Abstract] OR “Pathology, Speech”[Title/Abstract] OR “Speech Pathology”[Title/Abstract] OR “Speech Therapies”[Title/Abstract] OR “Therapies, Speech”[Title/Abstract] OR “Therapy, Speech”[Title/Abstract])

95 results 1.10.21
