# Supplementary material for: Health literacy interventions in adult speech and language therapy: A scoping review
Source: Health Expect. 2023 Sep 25;27(1):e13878. doi: 10.1111/hex.13878 (PMC10726155; doi:10.1111/hex.13878)
Supplement: Supplementary file 2 — Supporting information. [file HEX-27-e13878-s003.docx]

Supplementary material A

| **Study identifier, language and country** | **Participant demographics** | **Inclusion**  **criteria** | **Exclusion**  **criteria** | **Sampling / recruitment**  **methods** |
| --- | --- | --- | --- | --- |
| Atcherson et al., 2014  English; USA | N=101 audiology consumer articles (n=27 before 2011, n=74 after 2011); n= 124 SLT consumer articles (after 2007) from the public section of the ASHA website | Audiology documents on ASHA website (public section from 2010-2011) or SLT documents (from 2007) | Non audiology or SLT documents not available as information for the public within the stated timeframe Professional articles were not eligible | Unspecified purposive search of ASHA website |
| Azios et al., 2019  English; USA | N=43 websites (n=23 (53.5%) non-profit  organisation origin; n=15 (34.9%) commercial  origin; n=5 (11.6%) government origin) | Websites that provided information about aphasia treatment were included. Website origin was classified as commercial, non-profit organisation or government. | Websites that Google identified as advertisements, news, images and videos were excluded. | Systematic purposive online search based on key words identified by professionals and people with aphasia was employed across country-specific Google search engines |
| Dueppen et al., 2019  English; USA | N= 85 websites (n=31 (36.5%) non-profit organization origin; n=47 (55.3%) commercial origin; n=7 (8.2%) government origin) | Websites were included if they provided information directed to voice and voice disorders. | Websites that Google identified as advertisements, news, images, and videos were excluded. Websites that linked to scholarly peer-reviewed journal articles or books only were excluded. Websites with only navigation links and no substantive content, description, or explanation were not included. | Systematic purposive online search based on key words identified by SLT graduate students and faculty members working in SLT was employed across country-specific Google search engines |
| Eames et al., 2003  English; Australia | Patients: n=20 patients post-stroke (10 had aphasia and 10 did not) aged from 41-76 years of age (mean= 58 yrs; SD =9.2). Male= 14 ; female: 6. Education years range 5-17 (mean=11.4 yrs; SD=3). Mean years post stroke= 5.5 (SD 3.3). 15 has had 1 stroke; 3 had had 2 strokes and 2 had had 3 strokes.17 recruited from stroke support groups and 17 from aphasia-specific support group.    Caregivers: n=14 primary carers aged from 43-70 (mean=55.6 yrs; SD=7.4). Male=2 ; female=12. Years of completed education range 7-19 years. (mean= 12.9; SD=3.4). | To be eligible to participate, participants had to have had a stroke or be caring for someone who had a stroke, live in the community, and be able to read and speak English well enough to provide informed consent and participate in the study. | Candidates were ineligible if they were living in a residential facility; had an obvious cognitive impairment, inadequate vision, or hearing; or had global aphasia as assessed by the SLT involved in this study. | Convenience sampling of stroke survivors or informal carers living in the local community, attending university-based support groups, or known to the researcher |
| Ferreira & Figueiredo-Braga, 2019  English; Portugal | Phase 1: literature review Phase 2: Survey of a multidisciplinary panel of experts (n=4 SLTs; n=4 experts in clinical communication) Phase 3: focus group of patients and caregivers (n = 6). | Not stated | Not stated | Not stated |
| Hasselkus, 2009  English; USA | Unspecified number of studies related to topic included | Eligibility criteria for literature included not specified | Eligibility criteria for literature included not specified | Method for selecting included literature not specified |
| Hester & Benitez-McCrary, 2006  English; USA | Unspecified number of studies related to topic included | Eligibility criteria for literature included not specified | Eligibility criteria for literature included not specified | Method for selecting included literature not specified |
| Hester & Stevens-Ratchford  English; USA 2009 | Unspecified number of studies related to topic included | Eligibility criteria for literature included not specified | Eligibility criteria for literature included not specified | Method for selecting included literature not specified |
| Pothier et al., 2008  English; UK | N= 20 leaflets in routine clinical use in the Department of SLT in Gloucestershire Royal and Cheltenham General Hospital | Not stated | Not stated | Convenience sampling of leaflets in routine use within selected clinical sites |
| Rao, 2007  English; USA | Unspecified number of studies related to topic included | Eligibility criteria for literature included not specified | Eligibility criteria for literature included not specified | Method for selecting included literature not specified |
| Stefu, Slavych & Zraick, 2021  English; USA | N= 8 voice-related PROs chosen for readability analysis | PROM needed to be (1) related to voice disorders, (2) valid, (3) written in the English language, (4) linked to a publication source, and (5) able to be completed independently by the patient or a proxy | Not stated | Unspecified purposive search of databases to identify published voice-related PROs |
| von Wühlisch & Pascoe, 2010  English; South Africa | Case study 1: female aged 56 years, total laryngectomy, home language: Afrikaans, cannot read or write in any language; female SLT with 27 years of experience, working in tertiary hospital and speaking English as home language    Case study 2: caregiver (aged 33) to a child with a cleft palate, home language: Somali, no English literacy skills but proficient in Somali; female SLT with 14 years of experience, working in tertiary hospital and speaking English as home language    Case study 3: male aged 41, speech and swallowing difficulties, difficulties reading complex text, home language: Afrikaans;  female SLT with 2 years of experience, working in community clinic and speaking English and Afrikaans as home language    Case study 4: female aged 58, dysphonia, moderate literacy skills in English and Afrikaans, home language: Afrikaans; female SLT with 8 years of experience, working in tertiary hospital and speaking English as home language | Selection criteria for SLTs were that they had to be practising currently in the greater Cape Town area, and had to have at least 1 year’s post-qualification experience in managing at least one of the disorders that formed the focus of the study.    Selection criteria for clients were that they had to have one of the selected disorders or be a caregiver of a child with an unrepaired cleft lip and/or palate or a voice or swallowing disorder. They had to speak a language other than English as their first language because issues which might arise from the language divide between professionals and clients increase the potential for misunderstandings. Clients also had to have a poor socio-economic background and had to have low health and general literacy skills. | Not stated for SLTs;    Clients: excluded when they had any additional cognitive, psychological or intellectual disorders, as these were likely to affect their ability to give consent and their ability to express their opinion and perceptions. | Purposive sampling of SLTs practicing in the selected area and clients presenting with the conditions of interest |
| Zraick & Atcherson, 2012  English; USA | N= 12 English-language questionnaires chosen for readability analysis | Published PRO questionnaires related to dysphonia which were identified from leading dysphonia textbooks/book chapters, published reviews and scholarly and general internet searches. Questionnaires had to be linked to a publication source | Questionnaires that were designed to be administered in an interview or open-ended style were excluded. | Unspecified purposive search of dysphonia textbooks or book chapters, published reviews, and scholarly and general internet sources to identify published dysphonia-related PROs |
| Zraick, Atcherson & Brown, 2012  English; USA | N= 10 English-language questionnaires chosen for readability analysis | Published PROs related to stuttering. | Questionnaires that were designed to be administered in an interview or open-ended style were excluded. | Unspecified purposive search |
| Zraick, Atcherson & Ham, 2012  English; USA | N= 4 English-language questionnaires chosen for readability analysis | Published PRO questionnaires related to swallowing which were identified from leading swallowing textbooks/book chapters, published reviews and scholarly and general internet searches. Questionnaires had to be linked to a publication source | Questionnaires that were designed to be administered in an interview or open-ended style were excluded. | Unspecified purposive search of swallowing textbooks or book chapters, published reviews, and scholarly and general internet sources to identify published swallowing-related PROs |
