# Supplementary material for: Health literacy interventions in adult speech and language therapy: A scoping review
Source: Health Expect. 2023 Sep 25;27(1):e13878. doi: 10.1111/hex.13878 (PMC10726155; doi:10.1111/hex.13878)
Supplement: Supplementary file 3 — Supporting information. [file HEX-27-e13878-s004.docx]

Supplementary material B

| **Study identifier** | **Study Outcomes** | **Study Conclusion** |
| --- | --- | --- |
| Atcherson et al., 2014 | For the audiology-related section prior to 2011, the mean across readability formulas was a reading grade level of 11.2 (range: Grade 5 to graduate school). For the audiology-related section after 2011, the mean across readability formulas was a reading grade level of 10.5, (range: Grade 5 to graduate school). For audiology-specific changes before and after 2011, the FRE, F-K, and FOG formulas all demonstrated some improvement in readability. The FORCAST formula showed little to no change using either criteria (likely due to its formulaic limitation based only on the number of monosyllabic words). The correlations for the audiology section before 2011 were –0.65 for FRE–FORCAST (p = 0.0002) and 0.63 for FOG–FORCAST (p = 0.0004).  For the SLT-related section, the mean across readability formulas was a reading grade level of 11.0, (range: Grade 2 to graduate school). All but two readability formula pairs reached an alpha level less than 0.0001 using Pearson product moment correlations (r ≥ 0.70). All correlations suggest strong, positive agreement between the pairs of the different readability formulas.  A high proportion of articles exceeded the most stringent reading level for health information. FRE and F-K readability formulas showed some of the lowest proportion of articles exceeding either criteria compared to the other two readability formulas. | Many consumer articles on the ASHA website are likely to be of limited value to individuals with low health literacy. As a result, consumers might misinterpret or misapply information contained in the articles. |
| Azios et al., 2019 | Websites exhibited low quality with few websites obtaining Health On the Net (HON) certification or clear, thorough information as measured by the DISCERN. Readability scores were also poor. Approximate educational levels required to comprehend information on aphasia treatment websites ranged from 13 to 16 years of education. Significant differences were found between website origin and readability measures with higher levels of education required to understand information on websites of non-profit organizations. | The readability and quality of Internet information in aphasia are important concerns when considering Internet accessibility for people with aphasia. Future research is needed to understand how these obstacles influence the accessibility of Internet information for people with aphasia. |
| Dueppen et al., 2019 | Websites exhibited acceptable quality as measured by the DISCERN. However, only one website obtained the Health On the Net certification. Significant differences in quality were found among website origin, with government websites receiving higher quality ratings. Approximate educational levels required to comprehend information on the websites ranged from 8 to 9 years of education. Significant differences were found between website origin and readability measures with higher levels of education required to understand information on websites of nonprofit organizations. | Current vocal hygiene, vocal health, and prevention of voice disorders websites were found to exhibit acceptable levels of quality and readability. However, highly rated Internet information related to voice care should be made more accessible to voice clients through Health On the Net certification. |
| Eames et al., 2003 | Carers’ average reading ability was assessed at a grade 9 level or higher using the REALM. People with aphasia had considerably lower scores on the REALM, scoring between grades 4 and 6. Using the RIX formula, the readability levels of the 53 educational materials that were analyzed ranged from 6th grade to college level, with the majority (68%) being at or above a grade 9 level. Therefore, most of the materials used were written at a level that exceeded patient’s reading ability. As the reading difficulty of the materials decreased, participants’ satisfaction with them increased across the sample.  70% of the 53 materials analyzed in this study using the SAM scored as only adequate in terms of their content, literacy demand, graphics, layout and typography, learning stimulation and motivation, and cultural appropriateness. As the SAM score for select materials improved, participants’ satisfaction with them increased. People with aphasia had more specific design preferences than other participants (e.g. favoured the use of colour and addition of diagrams to support the text.) Over three quarters of participants in this study felt that their information needs were not met or only partially met during hospital admission. | The readability levels of written stroke educational materials were too high for many stroke survivors, particularly those with aphasia. Future research is needed to determine if the provision of written information tailored to individual needs in terms of its readability, design, and relevance makes a difference to clients’ and carers’ knowledge of and satisfaction with information about stroke. |
| Ferreira & Figueiredo-Braga, 2019 | The booklet includes sections addressing signs and symptoms of dysphagia individualized options of food consistency and preparation causes and complications of neurogenic dysphagia and how and when to contact health professionals. An appealing layout with illustrations facilitates the understanding of written information. | The use of booklets complements verbal patient-health professional interactions. In patients with neurogenic dysphagia it is expected to facilitate the understanding and recall of information and to respond to patients and family needs. |
| Hasselkus, 2009 | N/A given study design - “Before” and “After” examples of communicating clearly about health are provided. | Health care providers have a responsibility to make sure that the written and verbal information shared with patients is presented in a way that is accessible to understand and use. |
| Hester & Benitez-McCrary, 2006 | N/A given study design – resources provided to aid in the promotion of health literacy | SLTs and audiologists can add to communication aspects of health literacy by investigating the impact of communication disorders on health literacy and examining the relationship between existing health literacy assessments and speech-language and hearing. The need for SLTs and audiologists involvement in health literacy is clear and must be addressed via future research. |
| Hester & Stevens-Ratchford, 2009 | N/A given study design – conceptual frameworks regarding health literacy provided | There is a paucity of information available on health literacy within the SLT field. Health literacy research and intervention should be increased in this field. |
| Pothier et al., 2008 | The mean FRE score for the original leaflets was 59.5 and was 72.3 for the revised versions. This was highly statistically significant (p=0.006). The mean FKRGL for the original leaflets was 7.7 and was 5.4 in the revised versions (highly statistically significant (p=0.003)). The variability of readability outcome measures was lower in the revised group. Only 25% (n=5) of the original leaflets satisfied recommended levels of readability, while 75% (n=15) of the revised leaflets achieved these standards. Revised information leaflets were shorter | The readability of patient information leaflets can be improved by applying the guidelines in the NHS toolkit of producing patient information, but the use of passive sentences and difficult words in leaflets might not necessarily be reduced by using the NHS Toolkit. SLTs need to consider the readability of written information when designing and reviewing patient information leaflets. Readability statistics can be a useful tool in the process of leaflet design in SLT. |
| Rao, 2007 | N/A given study design – Steps to improve communication with patients provided | Patients need to be able to understand the implications of their diagnosis and the importance of prevention and treatment plans, and be able access health care services. This should not be adversely impacted because of communication problems. |
| Stefu, Slavych & Zraick, 2021 | At least 3 of the 4 formulas determined that 88% (n = 7) of the PROs exceeded the recommended 5th- to 6th -grade reading level. All 4 formulas determined that 75% (n = 6) of the PROs exceeded the recommended 5th - to 6th -grade reading level (minimum = 7.0, maximum = 16.8), with at least 1 formula revealing that 3 PROs were written at the college level (minimum = 12.6, maximum = 16.8). The FRE analysis showed that 100% (n = 8) of the PROMs were written at a comprehension level more difficult than the average 5th - to 6th grader can understand (minimum = 32 maximum = 88). The overall mean-reading grade level for the CLI, FOG, FORCAST, and SMOG metrics were 9th grade (readability score 9.25, SD 2.80), 9th grade (readability score 9.86, SD 3.29), 10th grade (readability score 10.30, SD 1.55), and 10th grade (readability score 10.51, SD 2.19), respectively. | Although awareness of health literacy has grown, voice-related PROs continue to be developed without full consideration of their reading grade level. Researchers should consider revising or developing PROs with consideration to reading grade level as well as other features to enhance readability. |
| von Wühlisch & Pascoe, 2010 | Management of three out of the four case studies was judged to be effective as determined by the SLTs’ and the clients’ accounts, as well as their ability to follow treatment guidelines. Various factors played a role in each particular case, but subjective opinions largely showed that the strategies assisted clients in maximizing health literacy and client recall, while ensuring an open channel of contact between clients and their SLTs post consultation. | This study provided insight into various strategies that are available for information exchange. Almost all the interventions that aim to improve compliance are complex and there is no one solution or universal tool for maximizing health literacy and recall. It depends on each client and their treating SLT which aspect of the combined strategies they see fit to use. It is important that SLTs consider health literacy and client recall issues in their everyday practice. |
| Zraick & Atcherson, 2012 | Results demonstrate that most voice-related PROs exceeded the 5th- to 6th -grade-reading levels recommended by health literacy experts. Based on the FORCAST formula, 3 of the PROs were written at the 7th grade reading level, 5 were written at the 8th-grade reading level, and the remaining 4 were written at the 9th grade reading level or higher. Readability varied widely across the 12 PROs. The mean FRE score was 79.1 (7th grade equivalent), the mean FOG score was 7.2, and the mean FORCAST score was 9. The PROM assessed to be “most readable” was The VoiSS (FRE= 89), while the “least readable” was judged to be the VAPP (FRE=66). | Currently existing PROM questionnaires may either need to be revised with psychometric properties re-analyzed or clinicians can take a number of different alternative approaches when a patient’s literacy skills are a possible concern. In the demand for standardization of voice-related PROM questionnaires, developers should consider readability as another testable construct, because poor readability may affect validity, reliability, and sensitivity. |
| Zraick, Atcherson & Brown, 2012 | Results demonstrate that many of the PROs exceeded the 5th to 6th grade reading levels recommended by health literacy experts. None of the questionnaires are written at the recommended reading grade level according to the FORCAST formula. The mean FRE score was 79 (7^th^ grade equivalent), the mean FOG score was 6.3, and the mean FORCAST score was 9.5. The PROM assessed to be “most readable” was The Communication Attitude Test (FRE= 100), while the “least readable” was judged to be the OASES-adult form (FRE=67). | The clinician should consider the average reading level needed to understand a particular PROM questionnaire when administering it to a patient or their proxy. Likewise, developers of PROM questionnaires should consider reading level of respondents and include information about this when reporting psychometric data. |
| Zraick, Atcherson & Ham, 2012 | All 4 swallowing-related PROs exceeded the 5th to 6th-grade reading levels recommended by health literacy experts. Based on the FORCAST formula, 1 of the PROM questionnaires was written at college reading level, 2 were written at the 10th-grade reading level, and 1 was written at the 9th-grade reading level. The two text-based variables that had the greatest predictability of reading grade level were the number of polysyllabic and hard words relative to the total number of words (FRE and FOG) and the number of monosyllabic words relative to the total number of words (FORCAST). Taken together, the higher the number of polysyllable or hard words, or the fewer the number of monosyllabic words the more difficult the PROM was calculated to read. The MDADI tool was consistently calculated to be most difficult to read by all three formulas. The other 3 PROM questionnaires (the EAT-10, the SWAL QOL, the SWAL CARE) were calculated to be roughly equivalent within each formula, but at several reading grade levels lower than calculated for the MDADI. | Currently existing PROM questionnaires may either need to be revised with psychometric properties re-analyzed or clinicians can take a number of different alternative approaches when a patient’s literacy skills are a possible concern. In the demand for standardization of swallowing-related PROM questionnaires, developers should consider readability as another testable construct, because poor readability may affect validity, reliability, and sensitivity. |
