# Supplementary material for: Health literacy interventions in adult speech and language therapy: A scoping review
Source: Health Expect. 2023 Sep 25;27(1):e13878. doi: 10.1111/hex.13878 (PMC10726155; doi:10.1111/hex.13878)
Supplement: Supplementary file 4 — Supporting information. [file HEX-27-e13878-s002.docx]

| Study Name | Study design - Cross-sectional study | | | | | | | | |
| --- | --- | --- | --- | --- | --- | --- | --- | --- | --- |
|  | Were the criteria for inclusion in the sample clearly defined? | Were the study subjects and the setting described in detail? | Was the exposure measured in a valid and reliable way? | Were objective, standard criteria used for measurement of the condition? | Were confounding factors identified? | | Were strategies to deal with confounding factors stated? | Were the outcomes measured in a valid and reliable way? | Was appropriate statistical analysis used? |
| Azios et al., 2019 |  |  |  |  |  | |  |  |  |
| Dueppen et al., 2019 |  |  |  |  |  | |  |  |  |
| Ferreira & Figueiredo-Braga, 2019 |  |  |  |  |  | |  |  |  |
| Atcherson et al., 2014 |  |  |  |  |  | |  |  |  |
| Green= yes; Red= no; Orange= unclear; Blue= N/A | | | | | | | | | |
|  |  |  |  |  |  |  |  |  |  |

Supplementary material C

Tool used: Moola S, Munn Z, Tufanaru C, Aromataris E, Sears K, Sfetcu R, Currie M, Qureshi R, Mattis P, Lisy K, Mu P-F. Chapter 7: Systematic reviews of etiology and risk . In: Aromataris E, Munn Z (Editors). JBI Manual for Evidence Synthesis. JBI, 2020. Available from [https://synthesismanual.jbi.global](https://synthesismanual.jbi.global/)

| Study Name | Study design - Quasi-experimental | | | | | | | | |
| --- | --- | --- | --- | --- | --- | --- | --- | --- | --- |
|  | Is it clear in the study what is the ‘cause’ and what is the ‘effect’ (i.e. there is no confusion about which variable comes first)? | Were the participants included in any comparisons similar? | Were the participants included in any comparisons receiving similar treatment/care, other than the exposure or intervention of interest? | Was there a control group? | Were there multiple measurements of the outcome both pre and post the intervention/exposure? | Was follow up complete and if not, were differences between groups in terms of their follow up adequately described and analyzed? | Were the outcomes of participants included in any comparisons measured in the same way? | Were outcomes measured in a reliable way? | Was appropriate statistical analysis used? |
| Pothier et al., 2008 |  |  |  |  |  |  |  |  |  |
| Green= yes; Red= no; Orange= unclear; Blue= N/A | | | | | | | | | |

Tool used: Tufanaru C, Munn Z, Aromataris E, Campbell J, Hopp L. Chapter 3: Systematic reviews of effectiveness. In: Aromataris E, Munn Z (Editors). JBI Manual for Evidence Synthesis. JBI, 2020. Available from <https://synthesismanual.jbi.global>

| Study Name | | | | | | | | | Study design – literature review | | | | |  |  |  |  |  |  |
| --- | --- | --- | --- | --- | --- | --- | --- | --- | --- | --- | --- | --- | --- | --- | --- | --- | --- | --- | --- |
|  | | | | | | | | | Is the review question clearly and explicitly stated? | Were the inclusion criteria appropriate for the review question? | Was the search strategy appropriate? | Were the sources and resources used to search for studies adequate? | Were the criteria for appraising studies appropriate? | Was critical appraisal conducted by two or more reviewers independently? | Were there methods to minimize errors in data extraction? | Were the methods used to combine studies appropriate? | Was the likelihood of publication bias assessed? | Were recommendations for policy and/or practice supported by the reported data? | Were the specific directives for new research appropriate? |
| Zraick & Atcherson, 2012 | | | | | | | | |  |  |  |  |  |  |  |  |  |  |  |
| Zraick, Atcherson & Ham, 2012 | | | | | | | | |  |  |  |  |  |  |  |  |  |  |  |
| Zraick, Atcherson & Brown, 2012 | | | | | | | | |  |  |  |  |  |  |  |  |  |  |  |
| Stefu, Slavych & Zraick, 2021 | | | | | | | | |  |  |  |  |  |  |  |  |  |  |  |
| Hester & Stevens-Ratchford, 2009 | | | | | | | | |  |  |  |  |  |  |  |  |  |  |  |
| Green= yes; Red= no; Orange= unclear; Blue= N/A | | | | | | | | | | | | | | | | | | | |
|  |  |  |  |  |  |  |  |  |  |  |  |  |  |  |  |  |  |  |  |
|  |  |  |  |  |  |  |  |  |  |  |  |  |  |  |  |  |  |  |  |

Tool used: Aromataris E, Fernandez R, Godfrey C, Holly C, Kahlil H, Tungpunkom P. Summarizing systematic reviews: methodological development, conduct and reporting of an Umbrella review approach. Int J Evid Based Healthc. 2015;13(3):132-40.

| Study Name | | Study design – opinion piece | | | | | | | | | | | |  |
| --- | --- | --- | --- | --- | --- | --- | --- | --- | --- | --- | --- | --- | --- | --- |
|  | | Is the source of the opinion clearly identified? | | | Does the source of opinion have standing in the field of expertise? | | Are the interests of the relevant population the central focus of the opinion? | | | Is the stated position the result of an analytical process, and is there logic in the opinion expressed? | | Is there reference to the extant literature? | Is any incongruence with the literature/sources logically defended? | |
| Rao, 2007 | |  | | |  | |  | | |  | |  |  | |
| Hester & Benitez-McCrary, 2006 | |  | | |  | |  | | |  | |  |  | |
| Hasselkus, 2009 | |  | | |  | |  | | |  | |  |  | |
| Green= yes; Red= no; Orange= unclear; Blue= N/A | | | | | | | | | | | | | | |
|  |  | |  |  | |  | |  |  | |  |  |  |  |
|  |  |  |  |  |  |  |  |  |  |  |  |  |  |  |

Tool used: McArthur A, Klugarova J, Yan H, Florescu S. Innovations in the systematic review of text and opinion. Int J Evid Based Healthc. 2015;13(3):188–195.

| Study Name | | | | Study design – Qualitative Study | | | | | | | | | |  |  |  |  |  |  |  |
| --- | --- | --- | --- | --- | --- | --- | --- | --- | --- | --- | --- | --- | --- | --- | --- | --- | --- | --- | --- | --- |
|  | | | | Is there congruity between the stated philosophical perspective and the research methodology? | | Is there congruity between the research methodology and the research question or objectives? | | Is there congruity between the research methodology and the methods used to collect data? | | | Is there congruity between the research methodology and the representation and analysis of data? | | Is there congruity between the research methodology and the interpretation of results? | | Is there a statement locating the researcher culturally or theoretically? | Is the influence of the researcher on the research, and vice- versa, addressed? | | Are participants, and their voices, adequately represented? | Is the research ethical according to current criteria or, for recent studies, and is there evidence of ethical approval by an appropriate body? | Do the conclusions drawn in the research report flow from the analysis, or interpretation, of the data? |
| Eames et al., 2003 | | | |  | |  | |  | | |  | |  | |  |  | |  |  |  |
| Green= yes; Red= no; Orange= unclear; Blue= N/A | | | | | | | | | | | | | | | | | | | | |
|  |  |  |  | |  | |  | |  |  | |  | | |  | |  |  |  |  |
|  |  |  |  |  |  |  |  |  |  |  |  |  |  |  |  |  |  |  |  |  |

Tool used: Lockwood C, Munn Z, Porritt K. Qualitative research synthesis: methodological guidance for systematic reviewers utilizing meta-aggregation. Int J Evid Based Healthc. 2015;13(3):179–187.

| Study Name | | | | | Study design – Case study | | | | | | | | | | | | | | |
| --- | --- | --- | --- | --- | --- | --- | --- | --- | --- | --- | --- | --- | --- | --- | --- | --- | --- | --- | --- |
|  | | | | | Were there clear criteria for inclusion in the case series? | | Was the condition measured in a standard, reliable way for all participants included in the case series? | | Were valid methods used for identification of the condition for all participants included in the case series? | | Did the case series have consecutive inclusion of participants? | Did the case series have complete inclusion of participants? | | Was there clear reporting of the demographics of the participants in the study? | | Was there clear reporting of clinical information of the participants? | Were the outcomes or follow up results of cases clearly reported? | Was there clear reporting of the presenting site(s)/clinic(s) demographic information? | Was statistical analysis appropriate? |
| von Wühlisch & Pascoe, 2010 | | | | |  | |  | |  | |  |  | |  | |  |  |  |  |
| Green= yes; Red= no; Orange= unclear; Blue= N/A | | | | | | | | | | | | | | | | | | | |
|  |  |  |  |  | |  | |  | |  |  | |  | |  |  |  |  |  |
|  |  |  |  |  |  |  |  |  |  |  |  |  |  |  |  |  |  |  |  |

Tool used: Munn Z, Barker T, Moola S, Tufanaru C, Stern C, McArthur A, Stephenson M, Aromataris E. Methodological quality of case series studies, JBI Evidence Synthesis, doi: 10.11124/JBISRIR-D-19-00099
